# Community Willingness to Participate in a Dengue Study in Aceh Province, Indonesia

**DOI:** 10.1371/journal.pone.0159139

**Published:** 2016-07-12

**Authors:** Harapan Harapan, Samsul Anwar, Aslam Bustaman, Arsil Radiansyah, Pradiba Angraini, Riny Fasli, Salwiyadi Salwiyadi, Reza Akbar Bastian, Ade Oktiviyari, Imaduddin Akmal, Muhammad Iqbalamin, Jamalul Adil, Fenni Henrizal, Darmayanti Darmayanti, Rovy Pratama, Jonny Karunia Fajar, Abdul Malik Setiawan, Allison Imrie, Ulrich Kuch, David Alexander Groneberg, R. Tedjo Sasmono, Meghnath Dhimal, Ruth Müller

**Affiliations:** 1 Medical Research Unit, School of Medicine, Syiah Kuala University, Banda Aceh, Indonesia; 2 Tropical Disease Centre, School of Medicine, Syiah Kuala University, Banda Aceh, Indonesia; 3 Department of Microbiology, School of Medicine, Syiah Kuala University, Banda Aceh, Indonesia; 4 School of Pathology and Laboratory Medicine, University of Western Australia, Nedlands, Western Australia, Australia; 5 Department of Statistics, Faculty of Mathematics and Natural Sciences, Syiah Kuala University, Banda Aceh, Indonesia; 6 Department of Biology, Faculty of Teacher Training and Education, Syiah Kuala University, Banda Aceh, Indonesia; 7 Department of Microbiology, Faculty of Medicine and Health Sciences, State Islamic University Maulana Malik Ibrahim, Malang, Indonesia; 8 Institute of Occupational Medicine, Social Medicine and Environmental Medicine, Goethe University, Frankfurt am Main, Germany; 9 Eijkman Institute for Molecular Biology, Jakarta, Indonesia; 10 Nepal Health Research Council (NHRC), Ministry of Health Complex, Kathmandu, Nepal; University of Texas at El Paso, UNITED STATES

## Abstract

**Background:**

Dengue virus infection is the most rapidly spreading vector-borne disease in the world. Essential research on dengue virus transmission and its prevention requires community participation. Therefore, it is crucial to understand the factors that are associated with the willingness of communities in high prevalence areas to participate in dengue research. The aim of this study was to explore factors associated with the willingness of healthy community members in Aceh province, Indonesia, to participate in dengue research that would require phlebotomy.

**Methodology/Principal Findings:**

A community-based cross-sectional study was carried out in nine regencies and municipalities of Aceh from November 2014 to March 2015. Interviews using a set of validated questionnaires were conducted to collect data on demography, history of dengue infection, socioeconomic status, and knowledge, attitude and practice regarding dengue fever. Two-step logistic regression and Spearman’s rank correlation (r_s_) analysis were used to assess the influence of independent variables on dependent variables. Among 535 participants, less than 20% had a good willingness to participate in the dengue study. The factors associated with good willingness to participate were being female, working as a civil servant, private employee or entrepreneur, having a high socioeconomic status and good knowledge, attitude and practice regarding dengue. Good knowledge and attitude regarding dengue were positive independent predictors of willingness to participate (OR: 2.30 [95% CI: 1.36–3.90] and 3.73 [95% CI: 2.24–6.21], respectively).

**Conclusion/Significance:**

The willingness to participate in dengue research is very low among community members in Aceh, and the two most important associated factors are knowledge and attitude regarding dengue. To increase participation rate, efforts to improve the knowledge and attitude of community members regarding dengue fever and dengue-related research is required before such studies are launched.

## Introduction

Dengue fever is a significant cause of morbidity and mortality especially among children, in several Asian and Latin American countries and has globally gained in epidemiological importance in recent decades [1] with estimates of annual dengue infections as high as 390 million and 128 countries reporting cases [2]. Community participation is vital for success in dengue control, dengue prevention and dengue research [3]. Initiatives prevention program in Latin Americas such as the integrated management strategy for dengue prevention and control (IMS-Dengue) and integrated vector management (IVM) required community participation [3]. A community based environmental management with community participation embedded in a routine control programme was effective as indicated by a significant reduction of dengue vectors in some regions [4,5]. Research on dengue also requires community participation in many ways. For example, active school absence based surveillance in Thailand [6,7] and school-based versus community-surveillance program in Peru [8] were completed because an adequate community participation. In addition, projects such as the establishment of biobanks, genetic and molecular epidemiology studies and dengue vaccine trials all depend on adequate numbers of participants. However, the recruitment may not be a trivial task. The difficulty in recruiting participants in clinical study is well documented [9–11] and this has resulted in cost increases, study completion and implementation delays, and statistical inconclusiveness [9–14]. Although general support of medical research seems high, less than half of individuals are willing to donate blood when a specific request for donation and storage is made [15].

Willingness to participate (WTP) surveys measure the willingness of research participants to take part in a particular study and the associated factors [16]. The WTP surveys have been conducted in various setting either in actual [17–20] or in hypothetical condition [18,21] and for non-infectious [20,22–25] or infectious diseases [21,26–29]. However, most of those studies did not require phlebotomy. A study found that the WTP rate in genetic research among patients after with myocardial infarction varied greatly between 24 US hospitals, from 40% to 100% [23]. In addition, another study found that public WTP to donate the blood for long-term storage and genetic research was 42% out of 3,130 research participants [30]. A study in South Korea found that only 25% of the research participants were willing to participate in clinical trials [31].

One factor that influences the WTP is the ethnic background of the participants. Studies found that African-Americans and White Americans differ in their WTP in medical research [19,32–34]. African-Americans were less likely to give a blood sample compared with White Americans [19,23,35]. Chinese participants were also less likely to donate blood for genetic research compared to non-Chinese participants [36]. A systematic review of ten clinical intervention studies revealed that Hispanics had a statistically significant higher WTP than non-Hispanic whites [37]. In addition, it is well accepted that motivations for research participation vary between participants of different cultural backgrounds [38,39]. Therefore an effort have been made to identify the factors that promote and hinder the WTP in clinical research among participants in the particular heritage [40]. As far to our best knowledge there is no study conducted in Indonesia to assess the WTP in particular in the dengue research setting.

Up to now, WTP has only very rarely been assessed for research on dengue. The only record we found, from Puerto Rico, assessed the interest to participate in a dengue vaccine efficacy trial [28] and revealed that barriers for participating in a vaccine trial included lack of information on dengue vaccine candidates, lack of trust in new vaccines and trial procedures, and fear of infection or other side effects.

In Indonesia, a dengue hyper-endemic country, all four dengue virus serotypes circulate in at least 433 regencies or municipalities (84.7%) and more than 200 million people are at risk of infection [41]. A recent nationwide study found that over half of children have been infected by the age of 5 [42]. The incidence of dengue fever has rapidly increased from 0.05 in 1968 to approximately 39.80 per 100,000 population in 2014 [41,43]. It is estimated that 30 million dengue infections occur in Indonesia annually [2] with approximately 1% case-fatality rate [44]. In Aceh, one of the high-prevalence provinces, there were 2,208 registered dengue cases in 2014 (46.6 per 100,000 population) [41]. In the wake of coordinated dengue prevention programmes planned for this province, this study aimed to determine the factors that influence WTP in dengue research that would require phlebotomy procedures among participating healthy community members.

## Material and Methods

### Study site and design

The cross-sectional study was conducted in nine regencies or municipalities of Aceh province, Indonesia (Aceh Besar, Aceh Selatan, Aceh Singkil, Aceh Tamiang, Aceh Tengah, Aceh Timur, Aceh Utara, Langsa and Sabang), from November 2014 to March 2015. Validated questionnaires from previous studies [45–48] were used in interviewing participants. The questionnaires covered demographic data and the history of dengue virus infection among participants and their family member(s), socioeconomic status (SES), and knowledge, attitude and practice (KAP) regarding dengue. Prior to use in the actual study, the questionnaires were tested for reliability among 30 participants in two regencies (Aceh Barat Daya and Aceh Pidie Jaya). Cronbach’s alpha 0.7 was used as the minimal cut-off for good internal consistency [49,50]. In addition, the normality of the data was analysed with the Kolmogorov–Smirnov test [51].

To represent the total population of Aceh (4,791,924 people in 2014) [52], 385 participants were required as the minimum sample size based on the assumption that 50% of the participants had a good WTP with a 5% margin of error and 95% confidence level [53]. The recruitment of participants was conducted using a stratified random sampling procedure. Nine out of 23 regencies and municipalities in Aceh were randomly drawn and participants were selected from these nine areas using judgmental sampling procedure. Higher numbers of participants were recruited from regencies with higher population numbers. Inclusion criteria were (1) healthy inhabitant, (2) age more than 16 years, (3) at least three months of residence in the current location, and (4) ability to communicate in Bahasa Indonesia.

### Measures

#### Dependent variable

The dependent variable consisted of behavioral intention as defined by WTP in a dengue study that requires phlebotomy. To ascertain WTP in the dengue study, participants were asked about their willingness to take part *and* to allow their family member to take part in a dengue study that requires phlebotomy and donation of 2.5 ml of their blood on the premise that they are infected with dengue virus. Each question had five possible answers in a Likert-like scale indicating agreement. The scoring for the Likert-like scale was: 5 = Very likely, 4 = Likely, 3 = Undecided, 2 = Unlikely; and 1 = Very unlikely. Higher scores reflected better WTP in the dengue study.

#### Independent variables

Demographic data of the participants including age, gender, educational attainment, type of occupation, religion, marital status, monthly income and type of residence were collected. Age was measured by date of birth and then converted into actual age. Education indicated the highest level of formal education completed. Occupation type was divided into five types including farmer, civil servant (working in the government sector), private employee (working in the private sector), entrepreneur (working as traders in traditional markets or owning small-scale businesses) and student or university student. Monthly income, the average of the money earned each month, was measured by asking the participants to choose the closest amount of money from a list. Type of residence was divided into rural and suburban areas. The history of dengue infection and having family member(s) who had suffered from dengue fever were collected based on participants’ recollections.

The socioeconomic status (SES) of participants was measured using an asset index based on Principal Component Analysis (PCA) [47]. The asset index was constructed based on the ownership of fifteen indicator assets and calculated as the sum of standardized asset scores multiplied by their respective factor loadings as proposed previously [48,54]. The quintiles of the asset index were calculated and the participants classified into 1^st^ (poorest) to 5^th^ quintile (least poor). The details indicator assets used are available in S1 Table.

To measure the knowledge of participants regarding the signs and symptoms of dengue fever and the transmission of dengue virus, 28 questions were adapted from previous studies [45,46]. Each correct answer from the possible responses (“yes” or “no”) was given a score of one, and incorrect answers were scored zero. There was no “do not know” option. To measure their attitude, participants were asked to respond to 15 questions from previous studies [45,46,48] on a five-point Likert-like scale indicating their agreement from ‘‘5 = strongly agree” to ‘‘1 = strongly disagree”. To assess preventive practices including the prevention of mosquito-man contact and elimination of mosquito breeding sites, 16 questions were adapted from previous studies [45,46]. Correct answers were given a score of one and incorrect ones, zero. Thus, higher KAP domain scores indicate better knowledge regarding dengue, more positive attitudes and better preventive practice. Details of questionnaires used for assessing KAP regarding dengue fever are available in S2 Table.

### Statistical analysis

The assessment of WTP and KAP domains was executed using a scoring system. The additive scale score ranged from 2 to 10 for the WTP and from 0 to 28, 15 to 75 and 0 to 16, respectively, for the three KAP domains. For each participant, scores for each question within the domain were summed up to obtain a single value. To assess the effect of independent variables on the dependent variable, two-step logistic regression analysis was used. For this, WTP and KAP were defined as”good” or”poor” based on an 80% cut-off point. In the first step, all independent variables were included in univariate logistic regression analysis. In the second step, since this study was exploratory in nature, all explanatory variables that were associated with WTP with a *P*-value ≤ 0.25 in the univariate analysis were entered into the multivariate logistic regression analysis [46,55]. The estimated odds ratio (OR) was interpreted in relation to one of the categories designated as the reference category. Confounding factors were explored by observing the difference between the crude OR from univariate analyses and the adjusted odds ratio (aOR) in multivariate analyses as proposed previously [46].

Additional analyses were conducted to assess correlations between WTP and some independent variables in the ratio scale data (SES and KAP scores) using Spearman’s rank correlation (r_s_). Spearman’s rank correlation was chosen because our data were not normally distributed as revealed by the Kolmogorov–Smirnov test. The confidence intervals for r_s_ were calculated as described previously [56]. All analyses were performed using Statistical Package for the Social Sciences software (SPSS for Windows, Version 15, Chicago, USA).

### Ethical approval

The study protocol was approved by the Institutional Review Board of the School of Medicine, Syiah Kuala University, Banda Aceh, Indonesia. The aims, risks, and benefits of the study were explained to each participant, and they were asked to sign a consent form prior to enrolment in the study. Participants were also informed that they could quit at any time during the interview session. After informed consent was obtained, the interviewers conducted the structured interviews. Participation in this study was voluntary and no incentive was given.

## Results

### Participant characteristics

In this study, 535 healthy community members from nine regencies and municipalities of Aceh province were surveyed. Their characteristics are summarized in Table 1. The average age of participants was 30.8 (17–70) years. The majority (66.9%) were living in suburban areas. No participant was illiterate. More than a third (38.7%) had completed senior high school (12 years) and approximately a fifth (27.9%) had a university degree. Half of the respondents earned less than 1 million Indonesian Rupiah (US $ 81) per month. Although only one-tenth of the participants declared having personally experienced dengue fever, one-fifth declared that family members had previously suffered from dengue fever. In addition, this study found that more than 60% of participants had poor knowledge regarding dengue and approximately 70% had poor attitude to dengue and poor preventive practices against dengue.

**Table 1:** Characteristics of participants and univariate logistic regression analysis showing predictors of willingness to participate (WTP) in a dengue study (good *vs*. poor) (*n* = 535).

Variable	n (%)	WTP score	WTP (Good/Poor)	Univariate logistic regression	
				OR (95% CI)	P–value
Regency					0.734
Aceh Tengah *(R)*	65 (12.1)	7.12±2.06	12/53	1	
Langsa	74 (13.8)	7.42±1.90	14/60	1.03 (0.44–2.42)	0.945
Aceh Besar	88 (16.4)	6.93±2.36	20/68	1.30 (0.58–2.89)	0.522
Aceh Utara	43 (8.0)	7.05±2.31	10/33	1.34 (0.52–3.44)	0.546
Aceh Singkil	49 (9.2)	7.12±2.02	9/40	0.99 (0.38–2.59)	0.990
Sabang	56 (10.5)	7.23±1.66	6/50	0.53 (0.18–1.52)	0.238
Aceh Timur	33 (6.2)	7.15±2.11	7/26	1.19 (0.42–3.38)	0.745
Aceh Selatan	67 (12.5)	6.36±2.40	10/57	0.77 (0.31–1.94)	0.586
Aceh Tamiang	60 (11.2)	7.43±1.95	14/46	1.34 (0.56–3.20)	0.503
Age group					0.273
17–29 *(R)*	289 (54.0)	6.97±2.26	63/226	1	
30–44	179 (33.5)	7.24±1.94	31/148	0.75 (0.47–1.21)	0.241
45–59	60 (11.2)	7.08±1.93	7/53	0.47 (0.20–1.09)	0.080
60–84	7 (1.3)	7.42±1.90	1/6	0.60 (0.07–5.06)	0.637
Sex					0.023*
Male *(R)*	166 (31.0)	6.97±2.07	22/144	1	
Female	369 (69.0)	7.13±2.14	80/289	1.81 (1.08–3.02)	
Education					0.109
Primary *(R)*	34 (6.4)	6.85±1.79	3/31	1	
Junior high school	31 (5.8)	6.39±2.14	1/30	0.34 (0.03–3.50)	0.368
Senior high school	207 (38.7)	6.93±2.18	39/168	2.40 (0.70–8.25)	0.165
Diploma	114 (21.3)	7.34±2.03	26/88	3.05 (0.86–10.80)	0.083
University graduate	149 (27.9)	7.28±2.13	33/116	2.94 (0.84–10.22)	0.090
Occupation					0.079
Farmer *(R)*	139 (26.0)	7.11±1.61	26/113	1	
Civil servant	83 (15.5)	7.29±1.97	20/63	2.84 (1.11–7.23)	0.029*
Private employee	100 (18.7)	7.01±2.38	21/79	3.91 (1.48–10.35)	0.006*
Entrepreneur	80 (15.0)	7.07±2.14	6/74	3.28 (1.25–8.57)	0.015*
Student/university student	133 (24.9)	6.88±2.33	29/104	3.44 (1.36–8.70)	0.009*
Religion					0.999
Muslim *(R)*	528 (98.7)	7.07±2.13	102/426	1	
Other	7 (1.3)	7.43±0.97	0/7	0.00 (0.00-NA)	
Marital status					0.204
Unmarried *(R)*	228 (42.6)	6.98±2.28	51/177	1	
Married	289 (54.0)	7.10±2.02	47/242	0.67 (0.43–1.05)	0.080
Widowed	18 (3.4)	8.0±1.08	4/14	0.99 (0.31–3.14)	0.989
Monthly income					0.137
<1 million *(R)*	270 (50.5)	7.02±2.16	56/214	1	
1—≤ 2 million	108 (20.2)	6.77±2.13	15/93	0.61 (0.33–1.14)	0.126
2—≤ 3 million	88 (16.4)	7.10±2.02	13/75	0.66 (0.34–1.28)	0.220
> 3 million	69 (12.9)	7.74±1.94	18/51	1.35 (0.73–2.49)	0.338
Type of residence					0.598
Suburb *(R)*	177 (33.1)	7.16±2.14	36/141	1	
City	358 (66.9)	7.04±2.11	66/292	1.13 (0.72–1.78)	
Have family member(s) suffered from dengue?					0.213
No *(R)*	117 (21.9)	7.12±2.30	27/90	1	
Yes	418 (78.1)	7.07±2.07	75/343	1.37 (0.83–2.26)	
Have you personally suffered from dengue?					0.275
No *(R)*	48 (9.0)	7.44±2.10	12/36	1	
Yes	487 (91.0)	7.04±2.12	90/397	1.47 (0.74–2.94)	
Socioeconomic level					0.293
Poorest quintile *(R)*	107 (20)	6.86±1.99	14/93	1	
2^nd^	107 (20)	6.90±2.18	22/85	1.71 (0.82–3.57)	0.147
3^rd^	107 (20)	6.91±2.15	18/89	1.34 (0.63–2.86)	0.444
4^th^	107 (20)	7.25±2.15	22/85	1.71 (0.82–3.57)	0.147
Richest quintile	107 (20)	7.48±2.07	26/81	2.71 (1.04–4.35)	0.038*
Knowledge regarding dengue fever					<0.001**
Poor *(R)*	341 (63.7)	6.82±2.07	41/300	1	
Good	194 (36.3)	7.54±2.12	61/133	3.36 (2.15–5.24)	
Attitude towards dengue fever					<0.001**
Poor *(R)*	381 (71.2)	6.68±2.06	39/342	1	
Good	154 (28.8)	8.06±1.94	63/91	6.07 (3.83–9.63)	
Preventive practice against dengue fever					0.018*
Poor *(R)*	377 (70.5)	6.96±2.11	62/315	1	
Good	158 (29.5)	7.37±2.11	40/118	1.72 (1.10–2.70)	

CI: confidence interval, OR: odds ratio, R: reference group

* Significant at 0.05

**Significant at 0.01

### Willingness to participate in dengue study and associated factors

Only 102 participants (19.1%) had a good WTP in the proposed dengue study. Univariate logistic regression analysis revealed that being female, working as a civil servant, private employee, entrepreneur or student/university student, having high SES, and good KAP domains were associated with good WTP (*P* < 0.05) (Table 1). Neither age group, education, religion, marital status, monthly income, types of residence nor previous personal or family experience with dengue fever were associated with WTP in dengue research.

Being female increased the odds of having a good WTP approximately twice. Compared to farmers, the odds of having a good WTP increased if the participants were working as civil servants (OR: 2.84; 95% CI: 1.11–7.23), private employees (OR: 3.91; 95% CI: 1.48–10.35) or entrepreneurs (OR: 3.44; 95% CI: 1.25–8.57). Participants grouped in the richest quintile had increased odds of having a good WTP compared to the poorest quintile (OR: 2.71; 95% CI: 1.04–4.35). As expected, the odds of having a good WTP were increased among participants who had good KAP domains (OR: 3.36 [95% CI: 2.15–5.24], 6.07 [3.83–9.63], 1.72 [1.72–2.70]). However, multivariate logistic regression analysis revealed that only knowledge and attitude towards dengue were independent predictors of WTP (OR: 2.30 [95% CI: 1.36–3.90] and 3.73 [2.24–6.21], respectively) (Table 2).

**Table 2:** Multivariate logistic regression analysis showing predictors of willingness to participate (WTP) in dengue study (good *vs*. poor) (*n* = 535).

Independent variable	Attitude to research	
	aOR (95% CI)	*P*–value
Sex		0.260
Male *(R)*	1	
Female	1.40 (0.77–2.54)	
Education		0.661
Primary *(R)*	1	
Junior high school	0.97 (0.21–4.52)	0.972
Senior high school	1.35 (0.29–6.34)	0.701
Diploma	1.43 (0.33–6.24)	0.633
University graduate	0.43 (0.04–4.81)	0.494
Occupation		0.558
Farmer *(R)*	1	
Civil servant	1.69 (0.48–5.95)	0.416
Private employee	1.41 (0.36–5.42)	0.621
Entrepreneur	2.39 (0.73–7.82)	0.149
Student/university student	1.63 (0.44–5.99)	0.462
Marital status		0.794
Unmarried *(R)*	1	
Married	1.66 (0.37–7.36)	0.504
Widowed	1.04 (0.53–2.06)	0.905
Monthly income		0.185
<1 million *(R)*	1	
1—≤ 2 million	1.13 (0.44–2.90)	0.791
2—≤ 3 million	0.54 (0.22–1.34)	0.183
> 3 million	0.54 (0.25–1.16)	0.117
Have family member(s) suffered from dengue?		0.145
No *(R)*	1	
Yes	1.56 (0.86–2.82)	
Knowledge regarding dengue fever		0.002*
Poor *(R)*	1	
Good	2.30 (1.36–3.90)	
Attitude towards dengue fever		<0.001**
Poor *(R)*	1	
Good	3.73 (2.24–6.21)	
Preventive practice against dengue fever		0.373
Poor *(R)*	1	
Good	1.28 (0.75–2.18)	

CI: confidence interval, aOR: adjusted odds ratio, R: reference group

* Significant at 0.05

**Significant at 0.01

Analysis with Spearman’s rank correlation confirmed that there was a weak association between SES and WTP (r_s_ = 0.09, *P* = 0.032) (Table 3). Spearman’s rank correlation analysis also confirmed that the strongest predictor associated with WTP in dengue research were attitude and knowledge regarding dengue with r_s_ = 0.49 and r_s_ = 0.19, respectively (*P* < 0.001), while preventive practice against dengue had a weak association (r_s_ = 0.13).

**Table 3:** Correlation between willingness to participate (WTP) in dengue research, socioeconomic status and knowledge, attitude and practice (KAP) regarding dengue (*n* = 535).

Variables	Correlation (95% CI)	*P*-value
Socioeconomic status—WTP	0.09 (0.02–0.16)	0.032*
Knowledge regarding dengue—WTP	0.19 (0.12–0.26)	<0.001**
Attitude regarding dengue—WTP	0.49 (0.43–0.54)	<0.001**
Preventive practice against dengue—WTP	0.13 (0.06–0.20)	<0.001**

CI: confidence interval

* Significant at 0.05

**Significant at 0.01

## Discussion

The recruitment of participants for medical research is complex and challenging [14,57,58], especially when studies are interventional or involve invasive sampling methods. Inability to recruit required numbers of participants within a defined geographical setting or timeframe may delay study completion and incur additional costs, or result in statistically inconclusive outcomes [12–14]. For example, a study found that only 31% out of 114 UK trials funded by two UK funding agencies (Medical Research Council and Health Technology Assessment) achieved original recruited target and 45% recruited <80% of their target and 53% of trials required an extension [14]. One of the reasons was fewer patients agreeing to participate than expected [14]. In contrast, there are studies that have been and continue to be successful in gaining the support and participation of the community in research on dengue for example active school absence–based surveillance for dengue in Thailand [6], hospital-based participation in Hawaii [59] and school-based versus community-surveillance program in Peru [8].

Several factors including site-specific dengue epidemiology and cultural characteristics of the study population are important for community participation [8]. Understanding the factors that are correlated with the WTP of a particular population is thus essential before one sets out to conduct research that requires the participation of the members of that community. The main aim of this study was to explore the extent of community support for dengue research in Aceh, Indonesia. This support was measured by participants’ stated WTP in dengue research that would involve donating 2.5 ml of their blood and allowing their family member(s) to do the same if infected with dengue virus. The findings reported here have both practical and public policy implications. From a practical perspective, they can be used to address modifiable factors that are correlated with poor WTP in order to increase participation. From a policy perspective, public participation in dengue research is important because it facilitates the evidence-based decision-making process and public acceptance of health policy derived there from. This can only be achieved if community members are willing to participate in research.

This is the first study conducted to specifically explore WTP in dengue research in Indonesia and its related factors. The first study regarding WTP in dengue-related research anywhere was reported from Puerto Rico where WTP in a dengue vaccine efficacy trial had been assessed [28]. Basically, the concept of our study is similar to those on willingness to donate a blood sample for genetic research. Therefore, the results can also be compared with those from such studies [18,20,23,35,36,40,60,61].

Our study revealed that the percentage of participants with good WTP in dengue research was very low, less than 20%. This willingness rate is much lower than those reported from European and North American countries (60 to more than 90%) [18]. However, these comparisons should be interpreted with caution because the willingness rate represents the percentage of respondents who had good WTP. The good WTP in the present study, however, is defined as a cumulative WTP rate between the willingness of the participants to donate their own blood *and* their willingness to allow their family member(s) to do the same if participants or their family member(s) suffered from dengue infection. In addition, “undecided” participants in our study were also classified as “poor WTP” because a cut-off point of 80% had been set. In Asian countries, Singapore for example, approximately only 50% of participants were willing to donate blood samples for genetic research [36]. Nationwide survey in South Korea found that 25% of the participants were willing to participate in clinical trials [31]. This may be an indication of differences in race, ethnicity or culture in the study participants [32–34,62].

In general the factors associated with WTP in dengue research were sex, occupation, SES, and KAP domains. The univariate analysis found increasing odds of being willing to participate in dengue research if participants were female, working as civil servant, private employee or entrepreneur, had high SES or good KAP domains. While in multivariate analysis revealed that only knowledge and attitude towards dengue were the independent predictors of WTP. The observed high WTP in dengue research among women may be associated with more pronounced altruism of women compared to men in Indonesia [63]. It is well known that altruism is one of the most important factors for WTP in a medical research study [40,64–67]. A previous study also found that male was associated with high refusal in a genetic study that required phlebotomy in US [20]. Interestingly, studies consistency found that gender had no association with WTP toward blood donation and storage for genetic research in US [30,68,69], Sweden [70] and in Singapore [36]. A study found that male however had higher WTP in cancer genetics research in US [71]. These inconsistency findings explain, in part, why gender was not a robust independent factor in the present study.

As expected, good knowledge and attitude regarding dengue were identified as positive predictors of WTP in dengue research. Our previous study revealed that there was a strong association between good attitudes regarding dengue and good preventive practices against dengue, and that there was a good translation of attitudes into preventive practice in Aceh [72]. Therefore, the high rate of WTP among participants who had a good attitude regarding dengue might reflect their efforts for dengue prevention. In addition, it has been reported that participants who have a high level of understanding (good knowledge) regarding a medical study are more likely to have a positive attitude towards participation in a medical study [73]. A study in South Korea found that good perception and high awareness concerning medical research were identified as positive predictors of WTP in medical research [31]. Our study revealed that good attitude regarding dengue was the strongest factor for good WTP. Similarly, a study on willingness to donate an organ found a significant positive correlation with attitude [74]. Studies found that good attitude toward genetic research [70] and donation and storage of blood specimens for genetic research [30] had strong association with high willingness to donate a blood sample for research in Sweden and US, respectively. In Singapore, attitude characteristic was also associated with willingness to donate blood for genetic study [36]. In addition, a good attitude toward clinical trials had strong association with WTP in medical research [31]. Furthermore, it has been well known that the lack of knowledge and awareness about medical study is a significant barrier to participate in medical study [31,75]. Altogether, this underscores that knowledge and attitude of participants are the cornerstones for their successful recruitment to a medical study. Therefore, higher enrolment rates could be achieved through positively changing individual knowledge and attitude toward dengue fever and also attitude toward participation dengue research studies such as a targeted educational approaches using an educational video [29].

It was expected that the experience of participants or their families with dengue fever would serve to increase a perception of one’s own vulnerability and therefore willingness to participate in research on the topic. For example, a study showed that knowing someone who suffered from a particular illness was a very strong motivator for participation in related medical research, stronger even than a personal history of that illness [16]. In addition, the history of genetic disease had strong correlation with the willingness to donate a blood sample in Sweden [70]. However, our study revealed that having a family member with a history of dengue fever or a personal history of dengue fever did not increase the WTP in dengue research. A similar finding was found from a couple studies in US [20] and Singapore [36] that family history of the disease had no association with willingness to donate blood for genetic research. A possible explanation from our previous study is that participants in Aceh who had personally suffered from dengue fever had no significantly better knowledge regarding dengue fever [72].

Surprisingly, in this study, formal education level was not associated with WTP in dengue research. A previous study also found that education was not an important factor for willingness to participate in medical research in USA [16] and or to donate blood for genetic research in some Singapore [36], US [20,35,68], and Sweden [70]. Furthermore, study also found that there was no association between education and WTP to participate in vaccine clinical trial in Asian countries such as China [29], India [21] and South Korean population [31]. One of the possible reasons is that, although formal education is associated with knowledge regarding dengue, it was not associated with attitude to dengue [72]. However, other studies did find that formal education was correlated with WTP in medical research [76,77].

Limitations of this study include that WTP is only a behavioral intention and therefore may not reflect or predict actual enrolment. Participants might be more likely to answer positively about WTP due to a cultural tendency to exhibit pleasing behavior. Second, WTP in dengue research may depend on factors other than demography and KAP regarding dengue. Therefore, factors not measured in this study (e.g., financial incentive, trust, altruism) could affect actual participation rates. Third, this study was unable to assess psychological barriers that might affect WTP in dengue research.

To increase the WTP in dengue or other medical research, various strategies can be used. First, efforts to increase the knowledge and attitude of community members regarding dengue and basic information related to a specific medical study are necessary. In addition, it is important to implement pre-consent education [78] during the enrolment of participants to enhance pre-existing knowledge and improve WTP. Second, (financial) incentives should be considered for increasing participation rates because this is a robust motivator for WTP [67]. In Singapore, healthcare-related incentives like free medical check-ups seemed more preferred than money [36]. In Indonesia, based on our dengue serosurveillance study (unpublished), we found that the participation was highly increased when incentives were offered. Therefore, an offer of incentives should be considered in dengue research study recruitment. Third, training for recruiters is also important and should include how to frame the study information in a positive way. Finally, if possible, the dengue research should be embedded in a government program because more community members may participate if the research is conducted by the government compared to other parties [36].

## Conclusion

This study found that the willingness to participate in dengue research was very low among community members in Aceh. The two most important associated factors are the knowledge and attitude of participants regarding dengue. Therefore, efforts to increase the knowledge and attitude regarding dengue in the communities prior to conducting dengue-related research may be critical for achieving sufficient participation rates.

## Supporting Information

S1 Table Indicator assets used for constructing the asset index (socioeconomic status) of participants. (PDF)

S2 Table The questionnaires used for assessing knowledge, attitude and practice regarding dengue fever. (PDF)
