# Supplementary material for: Community Willingness to Participate in a Dengue Study in Aceh Province, Indonesia
Source: PLoS One. 2016 Jul 12;11(7):e0159139. doi: 10.1371/journal.pone.0159139 (PMC4942142; doi:10.1371/journal.pone.0159139)
Supplement: S1 Table — (PDF) [file pone.0159139.s001.pdf]

S1 Table. Indicator assets used for constructing the asset index (socioeconomic status) of participants

Variables	Score
Piped-water	1
Toilets	1
Radio	1
Landline phone	1
Refrigerator	1
Personal computer	1
Bicycle	1
Motorcycle	1
Car	1
Internet connection	1
Own the housing unit	1
Having a separate room functioning as kitchen	1
The house is built with non-dirt flooring	1
The house is built with roof tiles	1
The house is built with brick walls	1
