# Supplementary material for: Community Willingness to Participate in a Dengue Study in Aceh Province, Indonesia
Source: PLoS One. 2016 Jul 12;11(7):e0159139. doi: 10.1371/journal.pone.0159139 (PMC4942142; doi:10.1371/journal.pone.0159139)
Supplement: S2 Table — (PDF) [file pone.0159139.s002.pdf]

S2 Table (A). Questions used for assessing the knowledge regarding dengue

Variables
Dengue is caused by a virus. *Yes*
Can all mosquitoes transmit dengue virus? *No*
Do the *Aedes* mosquitoes transmit dengue virus? *Yes*
Do flies transmit dengue virus? *No*
Do ticks transmit dengue virus? *No*
Does casual person to person contact transmit DF? *No*
Is dengue virus transmitted through food and water? *No*
Do mosquitoes breed in standing water? *Yes*
Do windows screens and bed net reduce mosquitoes? *Yes*
Do insecticides sprays (such as Baygon) reduce mosquitoes and prevent DF? *Yes*
Do tightly covering water containers reduce mosquitoes? *Yes*
Do mosquito repellents prevent mosquito bites? *Yes*
Does dengue virus transmitted to humans by the bite of female *Aedes* mosquitoes that have been infected? *Yes*
Is the rainy season the only season when DF present? *No*
Can you identify *Aedes* mosquitoes? *Yes*
When are the *Aedes* mosquitoes most likely to feed/bite? *Day time*
Is headache a symptom of DF? *Yes*
Is joint pain a symptom of DF? *Yes*
Is muscle pain a symptom of DF? *Yes*
Is pain behind the e*Yes* a symptom of DF? *Yes*
Is bone sore a symptom of DF? *Yes*
Are nausea/vomiting symptoms of DF? *Yes*
Is rash a symptom of DF? *Yes*
Is diarrhea common in DF? *Yes*
Is stomach pain common in DF? *Yes*
Is gums bleeding common in DF? *Yes*
Can a person be suffered from DF more than once? *Yes*
Does DF affect infants, children and adults? *Yes*

S2 Table (B). Questions used for assessing the attitude regarding dengue

Variables
Is DF a serious illness? *Strongly agree or Agree*
Are you at risk of getting DF? *Strongly agree or Agree*
Can DF be prevented? *Strongly agree or Agree*
Is controlling the breeding places of mosquitoes a good strategy to prevent DF? *Strongly agree or Agree*
Do you think that stagnant water around the houses in discarded tyres, broken pots and bottles are breeding places of *Aedes* mosquitoes? *Strongly agree or Agree*
Do you think communities should actively participate in controlling the vectors of DF. *Strongly agree or Agree*
Everyone has a chance to be suffered from DF. *Strongly agree or Agree*
If you experience signs and symptoms of DF, you would immediately come to Community Health Centre. *Strongly agree or Agree*
You are the key individuals in preventing DF. *Strongly agree or Agree*
All DF patients have a chance for a full recovery. *Strongly agree or Agree*
The government is doing their best to prevent DF. *Strongly agree or Agree*
Your neighborhood is a DF high-risk area. *Strongly agree or Agree*
Community members are capable of preventing DF. *Strongly agree or Agree*
You are capable of preventing DF. *Strongly agree or Agree*
Government actions are needed for DF prevention. *Strongly agree or Agree*

S2 Table (C). Questions used for assessing practice regarding dengue prevention

Variables
Use screen windows to reduce mosquitoes. *Yes*
Prevent water stagnation. *Yes*
Cut down bushes in the yard to reduce mosquitoes. *Yes*
Use mosquito eating fish to reduce mosquitoes. *Yes*
Cleaning of garbage/trash. *Yes*
Disposing water holding containers such as tires, parts of automobiles, plastic bottles, crack pots. *Yes*
Use of fan to prevent mosquito biting. *Yes*
Covering body with clothes when working in the bush, farm or forest. *Yes*
Cover water containers in the home. *Yes*
Change the water plant containers in the house every week. *Yes*
Change the water in flower containers. *Yes*
Check the waste / garbage that can block the flow of water around home. *Yes*
Participate in any of the dengue infection campaign in the community. *Yes*
Check and clean the drains / gutters roofs of the rainy season. *Yes*
Use bed net when sleeping during day. *Yes*
Frequently cleaning water filled containers and ditches around the house. *More than once per week*
